# Improvement of Lead Acetate-Induced Testicular Injury and Sperm Quality Deterioration by Solanum Anomalum Thonn. Ex. Schumach Fruit Extracts in Albino Rats 

**Published:** 2019-06

**Authors:** Samuel James Offor, Herbert Orji Mbagwu, Orish Ebere Orisakwe

**Affiliations:** 1 Department of Pharmacology and Toxicology, Faculty of Pharmacy, University of Uyo, UyoAkwa Ibom State, Nigeria; 2 Department of Experimental Pharmacology and Toxicology, Faculty of Pharmacy, University of Port-Harcourt, Rivers State, Nigeria; 3 World Bank Africa Centre of Excellence in Public Health and Toxicological Research (PUTOR), University of Port Harcourt, PMB,5323 Port Harcourt, Rivers State, Nigeria

**Keywords:** Testicular Toxicity, Lead Acetate, Solanum Anomalum, Oxidative Stress, Public Health

## Abstract

**Objective:** This study has investigated the protective role of a natural alternative, *Solanum anomalum* fruit extract in lead induced testicular toxicity in male albino rats.

**Materials and methods:** Twenty-four mature male albino rats were used, divided into four groups of six rats per group. Group 1 (control rats) were given distilled water (10ml/kg), group 2 received lead acetate solution 60mg/kg, group 3 received lead acetate (60mg/kg) followed by *Solanum anomalum* (452mg/kg) and group 4 rats were given lead acetate (60mg/kg) followed by *Solanum anomalum* (678mg/kg) by oral gavage daily for 28 days.

**Results:** Lead treated group showed significant increase in Malondialdehyde MDA (1.58 ± 0.09 to 1.90 ± 0.17 µmol/L of plasma) and decreases in Superoxide dismutase SOD, glutathione peroxidase (482.85 ± 53.43 to 247.18 ± 70.40 U/L of blood), total glutathione (1.11 ± 0.03 to 0.56 ± 0.31 ng/µL) (144.80 ± 7.00 and 122.39 ± 4.63 U/ml of blood), epididymal sperm reserve, testicular sperm count, % sperm motility and % sperm viability.

**Conclusion:** Co-administration of *Solanum anomalum* significantly reversed the effect of lead with restoration of histoarchitecture of the testes. *Solanum anomalum* may be a protective modulator of lead -induced testicular injury.

## Introduction

Lead has been implicated in various diseases (1). Exposure to lead may be through sources like leaded gasoline, lead smelting and coal combustion, lead-based paints, lead containing pipes or lead-based solder in water supply systems, battery recycling, grids and bearings, *etc. *(2). Other sources of lead include: ceramic glazes, toys, ammunition and jewelry and in some cosmetics and traditional medicines. In occupationally exposed, lead is known to reduce the seminal parameters including density, total sperm number and viability with increase in the number of pathological spermatozoa, decreased libido, altered spermatogenesis, chromosomal damage, infertility and changes in serum testosterone (3-5).

Male infertility accounts to about 50% of infertility cases in 10-15% of couples (6). One of the major factors associated with male infertility is the quantity and quality of sperm produced (7). Failure of spermatogenesis is the result of several causes such as systemic diseases, endocrine disorders, malnutrition, genetic factors and environmental hazards (8). Heavy metals may compromise male reproduction, as demonstrated by epidemiological and animal studies (9). Lead poisoning causes inhibition of testicular functions along with those of the secondary sexual glands like the prostrate, epididymis and seminal vesicle, resulting in the alteration of their biochemical composition and affecting both steroidogenesis as well as gametogenesis (10). Accumulation of lead in the testis is known to have anti-spermatogenic effect (11). According to Anjum *et al.* (12), the testis of lead treated rats revealed remarkable degeneration and atrophied seminiferous tubules with absence of regular differentiated stages of germ cells to mature spermatozoa.

Chelation with meso-2,3-dimercaptosuccinic acid (Succimer or DMSA) and D,L-2,3-dimercapto-1-propanesulfonic acid (Dimaval or DMPS); the 2,3-dimercaptopropanol (British Anti Lewisite, BAL or Dimercaprol), Ethylenediamine-tetracetic acid (EDTA), and D-penicillamine remain the mainstay of management of lead poisoning (13-15). 

 The chelation drug binds the molecules of lead aiding their excretion and subsequently reduction in the body burden of the lead (16). However, chelation drugs have some side effects. Succimer causes nausea, vomiting, diarrhea and skin rash; Penicillamine (abdominal pain, skin lesions, alopecia, stomatitis, glossitis, luecopenia, thrombocytopenia, enuresis); BAL (nausea, vomiting, sweating, high fever, hypertension, and tachycardia) (17-18, 14); DMPS causes transient increases in hepatic transaminase activity which however, resolves with discontinuation of drug(19); EDTA (Renal toxicity, cardiac problems due to hypocalcemia). Report exists of deaths due to treatment with EDTA. In addition to the adverse effects of these chelators, the prohibitive cost and scarcity of these agents pose serious management challenge in resource poor countries in the developing nations.

Fruits of the plant, *Solanum anomalum: *Thonn. Ex. Schumach is a plant, up to 2 meters in height with prickles up to 5mm long on stem, branches and midrib of the leaves (20). The fruit is a ball-shaped berry of 5-9 mm in diameter, shiny red when matureand green when young. The seeds are also globose 2-3 mm in diameter and ball-shaped (20). The mature red fruits of *Solanum anomalum*are harvested from the wild and made into soups and sauces, or can be eaten fresh. The exudate from the leaves and fruits is drunk or taken by enema 1-2 times daily as a treatment for leprosy in West Africa. In Nigeria, the fruit is used as a laxative and appetizer In a particularly village called Ikot Nta in Itumbonuso, Ini Local Government Area of Akwa Ibom State, a young Child with obvious symptoms of splenomegaly, known locally as*Ikpakup*was reported to have recovered fully by the mother after repeatedly eating the raw fruit every morning (21). The locals also lay claim to the fact that eating the raw fruits help to treat malaria. Also, the leaf exudate in treatment of gonorrhea and crushed fruits extracts are used as anti-inflammatory and analgesic (22). Apart from the above ethno-botanical and anecdotal uses, literature search revealed that very few scientific work has been done on the plant. This minor vegetable contains saponins, cardiac glycosides, anthraquinones, terpenes, flavonoids, tannins and alkaloids (21). The extract of *Solanum anomalum* show no acute or sub subacute toxicity up to 1000mg/kg in Wistar rats (Abubakar and Bisalla (23). Offor and Ubengama (21) determined the LD_50_ to be 2260 ± 131.78mg/kg and reported the antidiabetic effect of the ethanolic extract and fractions of the fruits. 

Reactive oxygen species (ROS) are usually generated when deleterious free radicals exceeds the body’s antioxidant defense machinery, a phenomenon referred to as oxidative stress. Oxidative damage cause direct cellular injury by inducing lipid and protein peroxidation and damaging of nucleic acids (24, 25). Organisms combat ROS accumulation through glutathione, glutathione peroxidase, superoxide dismutase and catalase (26-28). The balance between ROS production and antioxidant capacity plays a crucial role in the pathophysiology of diseases (29). 

Infection is believed to be the main cause of infertility in Nigeria. There have been reported cases of infertility after treatment of infection in Nigeria (30). There are higher rates of irreversible oligo- or azoospermia than most other causes of infertility in Nigeria (30). With lean resources, the management of infertility can be very challenging with huge societal implication. It is feared that exposure to environmental pollutants including heavy metals like lead may be responsible for more than 12 million infertile persons in Nigeria (30). Given the high cost, scarcity and wide range of adverse effects of chelators the classical antidotes of lead poisoning, continuous search for widely available ‘’natural antidotes’’ that will ameliorate or reverse the deleterious effects of lead in developing nations has been research focus in our laboratory. 

The present study seeks to examine the efficacy *Solanum anomalum* fruit extracts in mitigating Lead-induced oxidative stress and injury in the male reproductive system of male albino Wistar rats.

## Materials and methods

***Chemicals*** *: *Lead acetate trihydrate (May & Baker, England) was dissolved in deionized water. Thiobarbituric acid, eosin, formalin and hematoxylin (Merck, Germany); Superoxide Dismutase kit (Fortress Diagnostics Limited, UK); Glutathione peroxidase kit (Fortress Diagnostics Limited, UK); GlutathioneColorimetric Detection Kit for Plasma Total Glutathione (RayBiotech, Inc. USA); Rat ELISA (Enzyme-linked immunosorbent assay) kits (RayBiotech, Inc. USA and Assaypro LLC, USA) were used in this study.

***Animal husbandry:*** Twenty-four male albino Wistar rats, weighing 145 – 170 g (aged 11-15 weeks) obtained from the University of Uyo Animal house, were acclimatized for two weeks, maintained under controlled conditions of temperature (23 ± 2 °C) and humidity (50 ± 5 %) and a 12-h light–dark cycle, were used for the experiment (31). The animals were housed in sanitized polypropylene cages containing sterile paddy husk as bedding. The bedding of the cages was changed weekly, and the cages were cleaned as well. They had free access to standard rat pellet diet and water ad libitum (31). The procedures were performed according to the guidelines on the use of animals and approved by the Institutional Animal Ethical Committee of the University of Uyo, Nigeria (Ethical Approval No: UNIUYO/PHARM/2015/0153) (31).

***Plant collection:*** *Solanum anomalum* with the fruits was obtained from a farmland in ObotNdomItumbonuso village, Ini Local Government Area of Akwa Ibom State, Nigeria. It was identified and authenticated by Dr. M.E.Bassey, a plant taxonomist in the Department of Botany and Ecological Studies, University of Uyo. The plant specimen (voucher number UUH: No 75(a)) was deposited in the Herbarium of the Department of Pharmacognosy and Natural Medicine, Faculty of Pharmacy, University of Uyo, Nigeria (31).

***Preparation and Extraction:*** The fruits were separated from the stalk and air dried under room temperature for 3 weeks. The dried fruits were powdered using pestle and mortar. The extract was prepared by maceration (cold extraction) of 350.05g of the air-dried, powdered fruits of *S. anomalum* using 60% ethanol in distilled water (v/v) in an extracting jar. This set up was allowed to stand for 72 hours with occasional shaking. The extract was filtered, concentrated until constant weights were achieved and stored in a refrigerator at 2-8^o^C for use in subsequent experiments. This procedure was repeated 3 times for maximal extraction (yield 69.8%). The LD_50_ of *Solanum anomalum* was determined by Offor & Ubengama (21) as 2260 ± 131.78 mg/kg. The chosen doses were the middle dose (20% of the LD_50_) which is 452mg/kg and the high dose (30% of the LD_50_) which is 678 mg/kg.

***Experimental design:*** The rats were divided into four groups of six rats per group as follows (31):

Group 1: Control rats: They were given distilled water (10ml/kg) by oral gavage daily for 28 days.

Group 2: Contained lead acetate solution 60mg/kg by oral gavage daily for 28 days addition to standard feed and water (32). 

Group 3: Lead acetate (60 mg/kg) (32), plus *Solanum anomalum* fruit extracts (452 mg/kg) by oral gavage daily for 28 days (31).

Group 4: Lead acetate (60 mg/kg) (31), plus *Solanum anomalum* fruit extracts (678 mg/kg) by oral gavage daily for 28 days. In all animals received *Solanum anomalum* fruit extracts 90 mins after administration of lead (31).

At the end of the experiment and 24 hours after the last dose, animals were weighed and blood samples collected. 

***Blood sample collection*** *:* Blood sample was collected using the Orbital technique (33). Blood was collected from the retro-bulbar plexus of the medial canthus of the eye of the rat (34). The blood (without anticoagulant) was kept at room temperature for 30 minutes to clot. Afterwards, the test tube containing the clotted blood sample was centrifuged at 3,000 revolutions per minutes for 10 minutes. The clear serum supernatant was then carefully aspirated with syringe and needle and stored in a clean sample bottle at -20^O^c until use for biochemical assay.

***Determination of antioxidant levels in rat namely (*** 31 ***)*** *: *Superoxide dismutase (SOD) in whole blood using Superoxide Dismutase kit in accordance with manufacturer’s recommended protocols (Fortress Diagnostics Limited, UK).

    (i) Glutathione peroxidase (GSH-PX) in whole blood using Glutathione peroxidase kit in accordance with manufacturer’s recommended protocols (Fortress Diagnostics Limited, UK).

    (ii) Plasma Total Glutathione using RayBio^® ^GlutathioneColorimetric Detection Kit in accordance with manufacturer’s recommended protocols (RayBiotech, Inc. USA)

    (iii) Measurement of Malondialdehyde, MDA, a prototype of the thiobarbituric reactive substances (TBARS) as a biomarker of lipid peroxidation and oxidative stress using the modified thiobarbituric acid method (35). All assays were done in triplicates and the coefficient of variation was less than 3%.

***Determination of sperm characteristics:*** Epididymal sperm reserves, testicular sperm count, sperm motility and viability were determined according to the methods of Amann and Almquist (36); Carrel and Aston (37); Lasley *et al*., (38).

***Histopathology:*** The male rats were sacrificed under ether anaesthesia (39). The testes were excised, weighed, rinsed clean in saline, and preserved in 10% formalin for histopathological study (26). They were dehydrated serially through progressive concentrations of alcohol and cleared using xylene. After clearing, the tissues were embedded in paraffin wax and thin sections of about 5µm were made using the microtome. Each section was mounted on a clean glass slide and stained with Haematoxylin and Eosin. Later, a mounting medium (Canada balsam) was dropped on each tissue section and a cover slip placed on it and allowed to dry (40). They were examined with a light microscope. Photomicrographs were captured using a Moticam Images plus 2.0 (Motic China Group Ltd.) digital Camera attached to the microscope. 

***Statistical Analysis:*** Results were expressed as mean ± standard deviation, SD. Statistical analysis was carried out with one way analysis of variance (ANOVA) followed by Dunnette test. Values of p < 0.05 were considered to be significant.

## Results

The effect of *Solanum anomalum* (SA) on body weight, Relative weight of testis and sperm parameters on lead acetate-treated male albino Wistar rats is shown on Table 1. Lead acetate administration led to significant decrease in body weight (190.77 ± 10.95 to 164.37 ± 17.28g), relative weight of testis (0.69 ± 0.04 to 0.62 ± 0.05), epididymal Sperm reserve (39.57 ± 3.02 to 5.19 ± 2.25 x 10^6^), testicular sperm count (418.00 ± 24.58 to 250.77 ± 31.44 x 10^6^), percentage Sperm motility (85.00 ± 8.22 to 36.50 ± 8.07) and Viability (94.33 ± 2.50 to 87.33 ± 4.59) when compared with control group that received only distilled water.

Co-administration of *Solanum anomalum* (452 and 678 mg/kg) and lead acetate resulted in the significant reversal of the effect of the lead acetate. *Solanum anomalum* at these doses 452 and 678 mg/kg brought about the following dose dependent changes: the body weight (172.98 ± 18.01 and 194.53 ± 27.24g), relative weight of testis (0.68 ± 0.03 and 0.63 ± 0.03), epididymal Sperm reserve (14.45 ± 5.20 to 21.90 ± 8.28 x 10^6^), testicular sperm count (301.50 ± 59.28 and 323.74 ± 22.56x 10^6^), percentage Sperm motility (80.50 ± 2.95 and 83.17 ± 8.73) and Viability (89.00 ± 2.68 and 90.50 ± 2.51) respectively. 

**Table 1:** Effect of Solanum anomalum (SA) on Body weight, Relative Testis weight and sperm parameters in Lead acetate-treated male albino wistar rats

**Treatment**	**Body weight ** **(g)**	**Testis weight ** **(g/100g body ** **weight)**	**Epididymal ** **Sperm reserve** **(10** ^6^ **)**	**Testicular sperm ** **count (10** ^6^ **)**	**Percentage ** **Sperm ** **motility (%)**	**Viability (%)**
Distilled water (10ml/kg)	190.77 ± 10.95	0.69 ± 0.04	39.57 ± 3.02	418.00 ± 24.58	85.00 ± 8.22	94.33 ± 2.50
Lead Acetate (60mg/kg)	164.37 ± 17.28^a^	0.62 ± 0.05	5.19 ± 2.25^a^	250.77 ± 31.44^a^	36.50 ± 8.07^a^	87.33 ± 4.59^a^
Lead acetate (60mg/kg) + SA (452mg/kg)	172.98 ± 18.01	0.68 ± 0.03	14.45 ± 5.20^a^**,**^b^	301.50 ± 59.28^a^^b^	80.50 ± 2.95^b^	89.00 ± 2.68
Lead acetate (60mg/kg) + SA (678mg/kg)	194.53 ± 27.24	0.63 ± 0.03	21.90 ± 8.28^a^,^b^	323.74 ± 22.56^a^,^b^	83.17 ± 8.73^b^	90.50 ± 2.51

Data were expressed as mean ± SD.

a : significantly different when compared to the control group (p < 0.05);

b **:** significantly different when compared to the lead acetate- treated group (p < 0.05) (n = 6)

**Table 2:** Effect of Solanum anomalum (SA) on some Antioxidant and Lipid peroxidation parameters of Lead acetate-treated male albino wistar rats

**Treatment**	**Malondialdehyde** **(µmol/L of plasma)**	**Glutathione peroxidase ** **(U/L of blood)**	**Superoxide Dismutase ** **(U/ml of blood)**	**Total Glutathione** **(ng/µL)**
Distilled water (10ml/kg)	1.58 ± 0.09	482.85 ± 53.43	144.80 ± 7.00	1.11 ± 0.03
Lead Acetate (60mg/kg)	1.90 ± 0.17^a^	247.18 ± 70.40^a^	122.39 ± 4.63^a^	0.56 ± 0.31^a^
Lead acetate (60mg/kg) + SA (452mg/kg)	1.77 ± 0.10	436.86 ± 56.32^b^	130.51 ± 4.57^a^	1.01 ± 0.09^b^
Lead acetate (60mg/kg) + SA (678mg/kg)	1.62 ± 0.21^b^	454.11 ± 151.39^b^	134.36 ± 3.11^a^,^b^	1.22 ± 0.09^b^

Data were expressed as mean ± SD.

a : significantly different when compared to the control group (p < 0.05);

b **:** significantly different when compared to the lead acetate- treated group (p < 0.05) (n = 6).

Table 2 shows the effect of *Solanum anomalum* (SA) on Malondialdehyde, Glutathione peroxidase, total Glutathione and Superoxide Dismutase on Lead acetate-treated male albino Wistar rats. Lead acetate caused significant increase in the Malondialdehyde (1.58 ± 0.09 to 1.90 ± 0.17 µmol/L of plasma), and significant decrease in Glutathione peroxidase (482.85 ± 53.43 to 247.18 ± 70.40 U/L of blood), Total Glutathione(1.11 ± 0.03 to 0.56 ± 0.31 ng/µL) and Superoxide Dismutase(144.80 ± 7.00 and 122.39 ± 4.63 U/ml of blood).Co-administration of *Solanum anomalum* (452 and 678 mg/kg) and lead acetate resulted in the significant reversal of the effect of the lead acetate on these antioxidant parameters. *Solanum anomalum* at these doses 452 and 678mg/kg brought about the following dose dependent changes on these parameters: Malondialdehyde (1.77 ± 0.10 and 1.62 ± 0.21 µmol/L of plasma), Glutathione peroxidase (436.86 ± 56.32 and 454.11 ± 151.39 U/L of blood), Total Glutathione (1.01 ± 0.09 and 1.22 ± 0.09 ng/µL) and Superoxide Dismutase (130.51 ± 4.57 and 134.36 ± 3.11 U/ml of blood) respectively.

**Figure 1:**
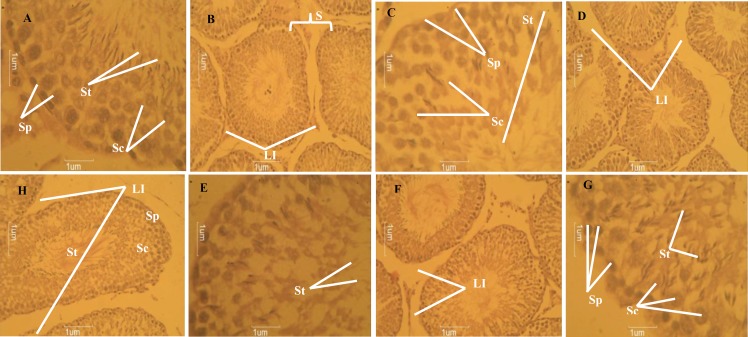
A. Histological photomicrograph of the testis stained with H&E techniques of Group 1 rats (control) that received distilled water 10ml/kg by oral gavage daily for 28 days showing seminiferous tubules with an abundant population of spermatogonia, spermatocJytes and spermatids (Mag. X400); B. Histological photomicrograph of the testis stained with H&E techniques of Group 1 rats (control) that received distilled water 10ml/kg by oral gavage daily for 28 days showing an abundance of Leydig cells-interstitial tissue cells (Mag. X 160); C. Histological photomicrograph of the testis stained with H&E techniques of Group 2 rats that received only Lead acetate 60mg/kg daily for 28 days showing low number of spermatogonia, spermatocytes and spermatids in the seminiferous tubules (Mag. X 400); D. Histological photomicrograph of the testis stained with H&E techniques of Group 2 rats that received only Lead acetate 60mg/kg daily for 28 days showing a depletion of Leydig cells-interstitial tissue cells in the testis. (Mag X 160). E. Histological photomicrograph of the testis stained with H&E techniques of Group 3 rats that received Lead acetate 60 mg/kg followed by Solanum anomalum (452 mg/kg) daily for 28 days showing seminiferous tubules with repopulated spermatids (Mag. X 400); F. Histological photomicrograph of the testis stained with H&E techniques of Group 3 rats that received Lead acetate 60 mg/kg followed by Solanum anomalum (452 mg/kg) daily for 28 days showing depletion of Leydig cells-interstitial tissue cells (Mag. X 160); G. Histological photomicrograph of the testis stained with H&E techniques of Group 4 rats that received Lead acetate 60mg/kg followed by Solanum anomalum (678 mg/kg) daily for 28 days showing seminiferous tubules with an abundant population of spermatogonia, spermatocytes and spermatids (Mag. X 400); H. Histological photomicrograph of the testis stained with H&E techniques of Group 4 rats that received Lead acetate 60 mg/kg followed by Solanum anomalum (678 mg/kg) daily for 28 days showing presence of only small pockets of Leydig cells-interstitial tissue cells (Mag. X 160).

***Histopathology of the testis:*** There was normal histoarchitecture of the testis in the control group (Figure 1 A & 1 B). Figs. 1 C & 1 D show the effect of lead acetate only on the testis. In the lead-acetate treated group, there was marked Leydig cell damage. There was also depletion of spermatogonia, spermatocytes and spermatids in the seminiferous tubules. The histomorphology of testis of lead acetate plus 425 mg/kg *Solanum anomalum *treated group is shown on Figures 1 E & 1 F. There was still depletion of Leydig cells-interstitial tissue cells and there was presence of seminiferous tubules with little or no spermatids lining side by side with those with spermatids, though all of them had spermatogonia. In the lead acetate plus 678mg/kg *Solanum anomalum *treated group, the small pockets of Leydig cells-interstitial tissue cells and the seminiferous tubules had an abundant population of spermatogonia, spermatocytes and spermatids Figures 1 G & H.

## Discussion

Testicular oxidative stress is the main feature in male infertility (41). Lead binds to human protamines during spermiogenesis, altering sperm chromatin stability and potentially affecting normal chromatin condensation (41, 42). Toxicant induced oxidative stress cause major damage to sperm quality by disrupting the anti-oxidant and reactive oxygen species (ROS) balance and thus resulting in abnormalities of spermatogenesis and male infertility (43, 44). Lead like most divalent metals is bound in tissues by ionic (in skeletal minerals) or coordination bonds and usually bound to albumin, enzymes, small peptides, cysteine, methionine, and selenomethionine (45). Lead binds to glutathione (GSH) and like other divalent metals can leave the cell to circulate in serum or lymph. The subsequent precipitous deposition of lead give rise to tissue or organ damage (46). It is believed that ROS have a detrimental effect on critical events on the steroidogenic pathway (47). Elevated levels of ROS elicit lipid peroxidation and membrane damage which lead to loss of sperm motility (48), inactivation of glycolytic enzymes and damage to the acrosomal membranes (49) which incapacitate the sperm cell (50).

S: Seminiferous tubule; Sc: Spermatocytes, Sp: Spermatogonia, LI: Leydig cells-Interstitial tissue cells; St: Spermatids

There has been continuous search for cheap and readily available phytochelators or natural antidotes with antioxidant properties that will ameliorate the deleterious effects of toxicants in man and animals. (51). Plant extracts have been reported to protect against lead induced toxicity in experimental animals. Some of these include: Sesame oil (52), the leaves of *Moringa oleifera *(53), Grape seed extract, *Vitisvinifera* (54), aqueous garlic extract (55), latex of* Ficuscarica *(56), methanolic extract of *Pongamiapinnata* flower (57) and many others.

Tissue MDA level is a vital diagnostic parameter for the determination of oxidative stress as it is one of the products of peroxidized polyunsaturated fatty acids PUFA (58). The increased level of MDA suggests an increase in lipid peroxidation (58, 59). Heavy metals are known to increase MDA levels in rat tissues (58, 60-61). In the present study, MDA level increased in the lead acetate treated group suggesting a lipid peroxidation effect of lead. The increased level of MDA suggests the generation of lipid peroxides, loss of membrane structure and function. The observations in this study are in harmony with the increase in testicular MDA levels in lead-treated rats relative to the control group (58, 59). In this study *Solanum anomalum*significantly decreased the MDA level in lead acetate treated rats. In another study blood antioxidant parameters SOD, GSH, CAT showed parallel relationship with seminal parameters (62). A positive correlation was also seen between blood MDA with percentage abnormal morphology and dead sperms (62). These reports are similar to our observations on the effects of SA on blood antioxidant and seminal parameters in lead acetate treated rats. 

Glutathione is metal chelator involved in cellular response, transport, and excretion of cations and is a biomarker for toxic metal overload (63). Chelators mobilize metals from tissues and maintain the chelate moiety during circulation to the kidneys for excretion in the urine, and to the liver for excretion in the bile (19). Cells possess some protective mechanisms against the damaging effects of reactive oxygen species ROS. The superoxide dismutase, SOD mops up superoxide radical converting it into H_2_O_2_ and eventual rapid conversion to water by catalase CAT or glutathione peroxidase GPx (64). Furthermore, glutathione peroxidase reduces lipid hydroperoxides to alcohols. Inhibition of any these antioxidant enzymes may lead to deleterious effects due to accumulation of superoxide radicals and hydrogen peroxide (64). Our study shows that the activities of antioxidant enzymes, superoxide dismutase and glutathione peroxidase in testis were restored to normal level by *Solanum anomalum*adminstration to lead acetate treated rats (65, 66). This observation in addition to the reduction in Malondialdehyde, MDA level in the *Solanum anomalum *treated groups, suggests a reactive oxygen species ROS-scavenging activity of *Solanum anomalum.*

The spermatozoa membranes are predominantly polyunsaturated fatty acids hence are susceptible to ROS attack and lipid peroxidation (67, 68) following exposure to lead. Oxidative stress represents an imbalance between the production of free radicals and the biological system's ability to readily detoxify the reactive intermediates or to repair the resulting damage (31, 69). It has been reported as a major mechanism of lead induced toxicity (31, 70). Under the influence of lead, onset of oxidative stress occurs on account of two different pathways occurring simultaneously. Firstly, the generation of reactive oxygen species, ROS and secondly, the antioxidant reserves become depleted (71). In addition to targeting the sulfhydryl SH groups, lead Pb^2+^ by molecular mimicry can replace the zinc ions Zn^2+^ an important co-factor of these antioxidant enzymes and inactivate them (72). Lipid peroxidation may also bring about membrane damage leading to decreased in sperm motility by a rapid loss of intracellular ATP, an increase in sperm morphology defects and acrosomal damage (41). Some active components in *Solanum anomalum* tend to mop up reactive oxygen radicals from lead acetate exposure, decrease lipid peroxidation and increase the activity of antioxidant enzymes (64). These properties of *Solanum anomalum* may play a positive role in the defense against oxidative stress induced by lead acetate. 

Lead acetate-mediated toxicity resulted in a significant decrease in epididymal sperm reserve, testicular sperm count, percent sperm motility and percent sperm viability (73). Similar observation was reported by Shan *et al*., (74); Falana and Oyeyipo (75). Most of these parameters were increased as a result of the protective effects of *Solanum anomalum*, especially with the higher dose of 678mg/kg. Lead accumulates preferentially in the epididymis and other accessory glands and Leydig cells appear to be its primary target (76). There was a depletion of Leydig cells (that secrete testosterone)-interstitial tissue cells in the testis of rats treated with lead acetate as well as depleted population of spermatogonia, spermatocytes and spermatids. Testis of rats treated with 678 mg/kg *Solanum anomalum* also had small pockets of Leydig cells-interstitial tissue cells but was higher than what was observed in the testes of rats treated with lead acetate only, in addition to an abundant population of spermatogonia, spermatocytes and spermatids. This protective effect of *Solanum anomalum*on the testis may be attributed to some of the active ingredients contained in the fruits of *Solanum anomalum*such as flavonoids, saponins, terpenes, tannins and alkaloids. Lead had been reported to cause hypospermia, lowered testosterone levels and testicular atrophy in male lead battery workers (77). The histopathological changes of lead on the testis include necrosis in seminiferous tubules, degenerative changes and edema in interstitial tissue (58). Lead acetate induced severe testicular toxicity as shown in the histopathology. These histopathological changes of lead on the testis namely separating of cells from basal region, edema in interstitial tissue, degenerative changes in seminiferous tubules and decreasing number of spermatogenic cells were associated with marked changes of biochemical parameters. Administration of *Solanum anomalum* in the lead acetate treated rats resulted in the restoration of the normal histoarchitecture of the testis.

## Conclusion

Taken together, the dose dependent reversal effect of *Solanum anomalum* extract on MDA, antioxidant biomarkers and histopathological alterations in the rat testis following lead acetate administration suggest a beneficial effect which may be extrapolated to man. Hence, it might be postulated that the ameliorative effect of *Solanum anomalum* in this study could be attributed to its rich antioxidants principles and ROS scavenging effect.
